# Validity and Impact of Methods for Collecting Training Data for Myoelectric Prosthetic Control Algorithms

**DOI:** 10.1109/TNSRE.2024.3400729

**Published:** 2024-05-22

**Authors:** Troy N. Tully, Caleb J. Thomson, Gregory A. Clark, Jacob A. George

**Affiliations:** Department of Biomedical Engineering, The University of Utah, Salt Lake City, UT 84132 USA; Department of Biomedical Engineering, The University of Utah, Salt Lake City, UT 84132 USA; Department of Biomedical Engineering, The University of Utah, Salt Lake City, UT 84132 USA; Department of Electrical and Computer Engineering, the Department of Physical Medicine and Rehabilitation, the Department of Biomedical Engineering, and the Department of Mechanical Engineering, The University of Utah, Salt Lake City, UT 84132 USA

**Keywords:** Prosthetic control, neuroprostheses, myoelectric prostheses, brain-computer interfaces, prosthetic hand, machine learning, training data, EMG

## Abstract

Intuitive regression control of prostheses relies on training algorithms to correlate biological recordings to motor intent. The quality of the training dataset is critical to run-time regression performance, but accurately labeling intended hand kinematics after hand amputation is challenging. In this study, we quantified the accuracy and precision of labeling hand kinematics using two common training paradigms: 1) mimic training, where participants mimic predetermined motions of a prosthesis, and 2) mirror training, where participants mirror their contralateral intact hand during synchronized bilateral movements. We first explored this question in healthy non-amputee individuals where the ground-truth kinematics could be readily determined using motion capture. Kinematic data showed that mimic training fails to account for biomechanical coupling and temporal changes in hand posture. Additionally, mirror training exhibited significantly higher accuracy and precision in labeling hand kinematics. These findings suggest that the mirror training approach generates a more faithful, albeit more complex, dataset. Accordingly, mirror training resulted in significantly better offline regression performance when using a large amount of training data and a non-linear neural network. Next, we explored these different training paradigms online, with a cohort of unilateral transradial amputees actively controlling a prosthesis in real-time to complete a functional task. Overall, we found that mirror training resulted in significantly faster task completion speeds and similar subjective workload. These results demonstrate that mirror training can potentially provide more dexterous control through the utilization of task-specific, user-selected training data. Consequently, these findings serve as a valuable guide for the next generation of myoelectric and neuroprostheses leveraging machine learning to provide more dexterous and intuitive control.

## I. Introduction

The current standard of care for upper-limb amputees is unsatisfactory, and, as a result, up to 50% of upper-limb amputees abandon their prostheses, citing poor and unreliable control as a primary reason [1], [2]. One approach to providing more intuitive and reliable control is to leverage supervised machine-learning algorithms that correlate residual muscle activity to motor intent. These supervised machine-learning algorithms require a training session in which a patient-specific training dataset is collected. The training dataset consists of synchronized muscle activity and the intended kinematic positions of the phantom limb.

**Fig. 1.**
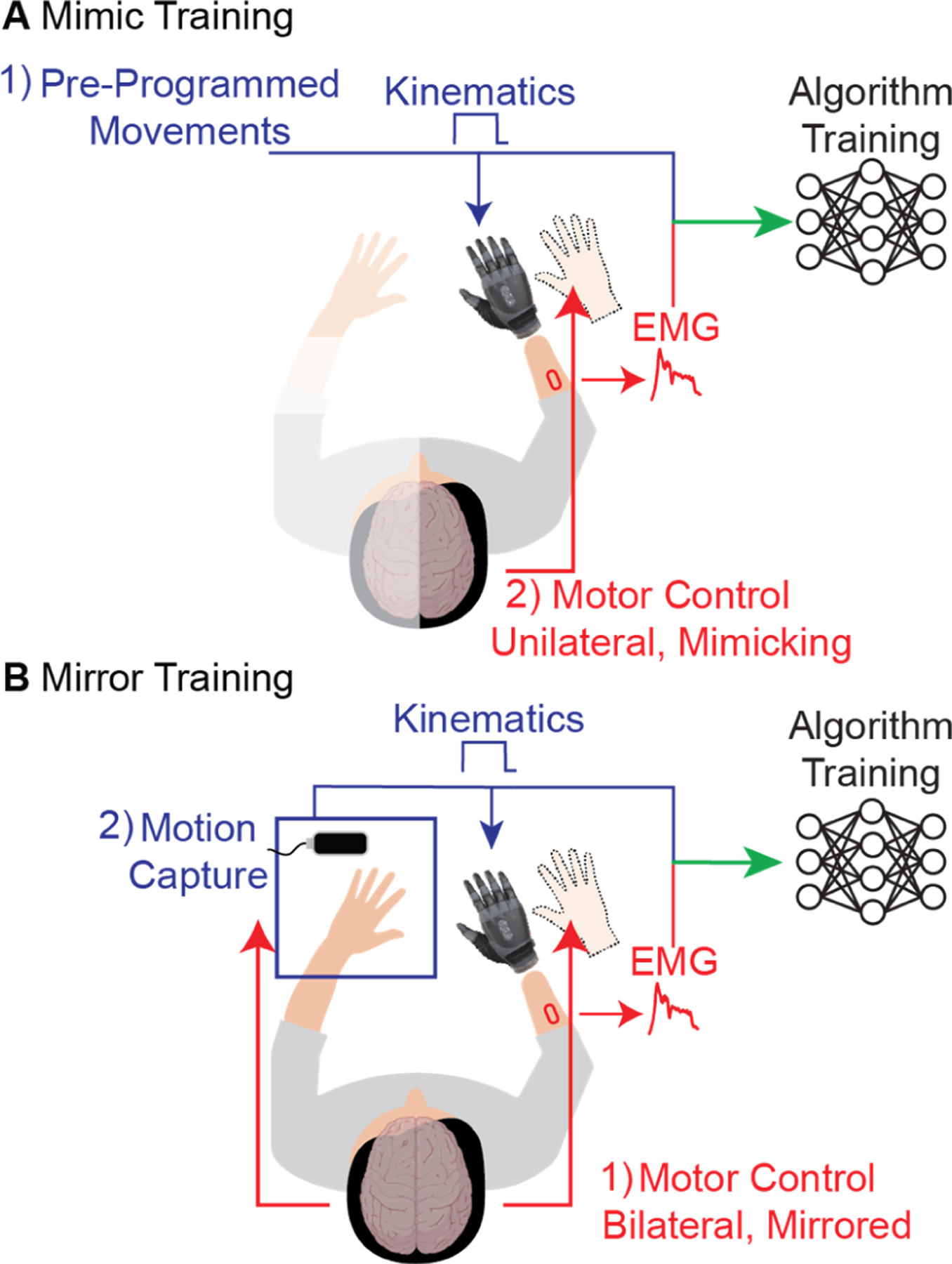
Two approaches to collecting training data for supervised machine-learning algorithms for myoelectric prostheses. A) With mimic training, the user watches a prosthesis move through pre-programmed movements and attempts to mimic the movement of the prosthesis with their phantom limb. The resulting training dataset consists of EMG from the residual muscles of the phantom hand and the pre-programmed kinematics of the prosthesis. B) With mirror training, the user performs bilaterally mirrored movements, such that the motion of their intact contralateral hand mirrors that of their phantom limb. The resulting training dataset consists of EMG from the residual muscles of the phantom hand and the mirrored kinematics of the contralateral limb (determined by motion capture).

To date, most research has focused on improving the machine-learning algorithm [3], [4], [5], [6], [7], [8], [9], [10]. However, the quality of the training data is also a critical component of the run-time performance of machine-learning algorithms [3], [4], [11]. Two widely used approaches (i.e., training paradigms) for collecting training data for prostheses exist. One training paradigm, herein referred to as “mimic training,” relies on amputees’ mimicking preprogrammed movements of a prosthesis with their missing hand such that the corresponding muscle activations are correlated to preprogrammed kinematics of the prosthesis (Fig. 1A) [12], [13], [14], [15], [16], [17]. This training method is most popular due to its low cost, ease of use, and availability. A second training paradigm, herein referred to as “mirror training,” relies on unilateral amputees’ mirroring their contralateral hand with their missing hand such that the muscle activations of the missing hand are correlated to the kinematics of their intact contralateral hand determined via motion capture (Fig. 1B) [18], [19], [20], [21], [22]. With the advent of low-cost motion capture, this training paradigm has become more popular in recent years. In this study, we directly compared these two training paradigms in terms of their ability to label intended kinematics and the resultant prosthetic control. We focus strictly on regression-based prosthetic control algorithms, for which the spatiotemporal accuracy and precision of the training data are particularly important.

Under mimic training, motor intent is most commonly determined by assuming that the amputee participant is perfectly mimicking the predetermined motion of a prosthesis with their missing hand. However, the validity of this assumption is unclear. There is at least some uncertainty in the temporal alignment of the predetermined and mimicked motions due to participant reaction time. Indeed, algorithms often preprocess the training data by aligning the preprogrammed kinematics with Electromyography (EMG) features in order to account for temporal delays [3], [7], [23]. Furthermore, mimic training also assumes that the user performs perfectly identical kinematic motions while mimicking the prosthesis, and this assumption is likely invalid due to the natural variance in hand motion.

The alternative paradigm, mirror training, has the potential to capture natural variance in both kinematic range and timing. But mirror training is founded on its own assumption that an individual can more accurately mirror their own contralateral hand than the preprogrammed motion of a prosthesis. Here, we sought to answer this question explicitly, using data from non-amputee participants where the ground-truth kinematics of the prosthesis, target hand, and contralateral hand could all be recorded simultaneously. We show that mimic training fails to account for biomechanical coupling and temporal changes in hand posture, and that mirror training results in more accurate and precise training data that yields greater machine learning accuracy.

Finally, using a small cohort of unilateral transradial amputees, we directly compared mimic and mirror training on the clothespin relocation task of functional upper-limb dexterity [24]. We show that mirror training results in significantly faster task completion speeds than does mimic training. Altogether, this work highlights the importance of accurate training data in the run-time performance of prostheses. As the prevalence of regression control grows in the field of prostheses, this work can serve as a basis for identifying, understanding, and correcting assumptions and errors in prosthetic training data, thereby leading to more dexterous and intuitive prostheses.

## II. Methods

### A. Human Subjects

Four transradial amputees and 13 healthy, non-amputee participants were recruited for this study. All participants were between the age of 18 and 65 years old. The four transradial amputees all had prior myoelectric experience. Informed consent and experimental protocols were carried out in accordance with the University of Utah Institutional Review Board.

### B. Signal Acquisition

Hand kinematics were tracked using an infrared motion capture camera (Leap Motion, Ultrahaptics, Bristol, UK). Custom MATLAB software was used to convert vectors into joint angles using an orthogonal vector of the palm and a vector from the user’s index finger. Joint angles were calculated for flexion/extension of D1–D5, abduction/adduction of D1, flexion/extension of the wrist, and pronation/supination of the wrist. After data collection, all joint angles were normalized post-hoc from −1 (maximum extension/supination) to 0 (rest), and from 0 to 1 (maximum flexion/pronation). The rest position of each joint was determined per participant as the average angle recorded over a 15-second rest period at the beginning of each training session.

For the healthy participants, EMG was recorded in synchrony with the hand kinematics using a custom sleeve with 32 embedded surface electrodes [25]. For the amputee participants, EMG was recorded from the surface of the residual limb using 32 adhesive electrodes (Cardinal Health, Kendall, H124SG, Clinton Township, MI, USA) placed under a functional-test socket [26]. EMG was sampled at 1 kHz using Micro2+Stim Front-Ends and a Summit Interface Processor (Ripple Neuro LLC, Salt Lake City, UT, USA). The 300-ms smoothed Mean Absolute Value (MAV) was calculated at 30 Hz for the 32 electrodes, as well as for all possible differential pairs (i.e., 496 differential pairs). The final acquired data consisted of the EMG MAV from 528 channels (32 single-ended electrodes + 496 differential pairs) and joint angles from the eight joints listed above.

### C. Data Collection and Training Data

#### 1) Biomechanical Analysis With Healthy Participants:

**Fig. 2.**
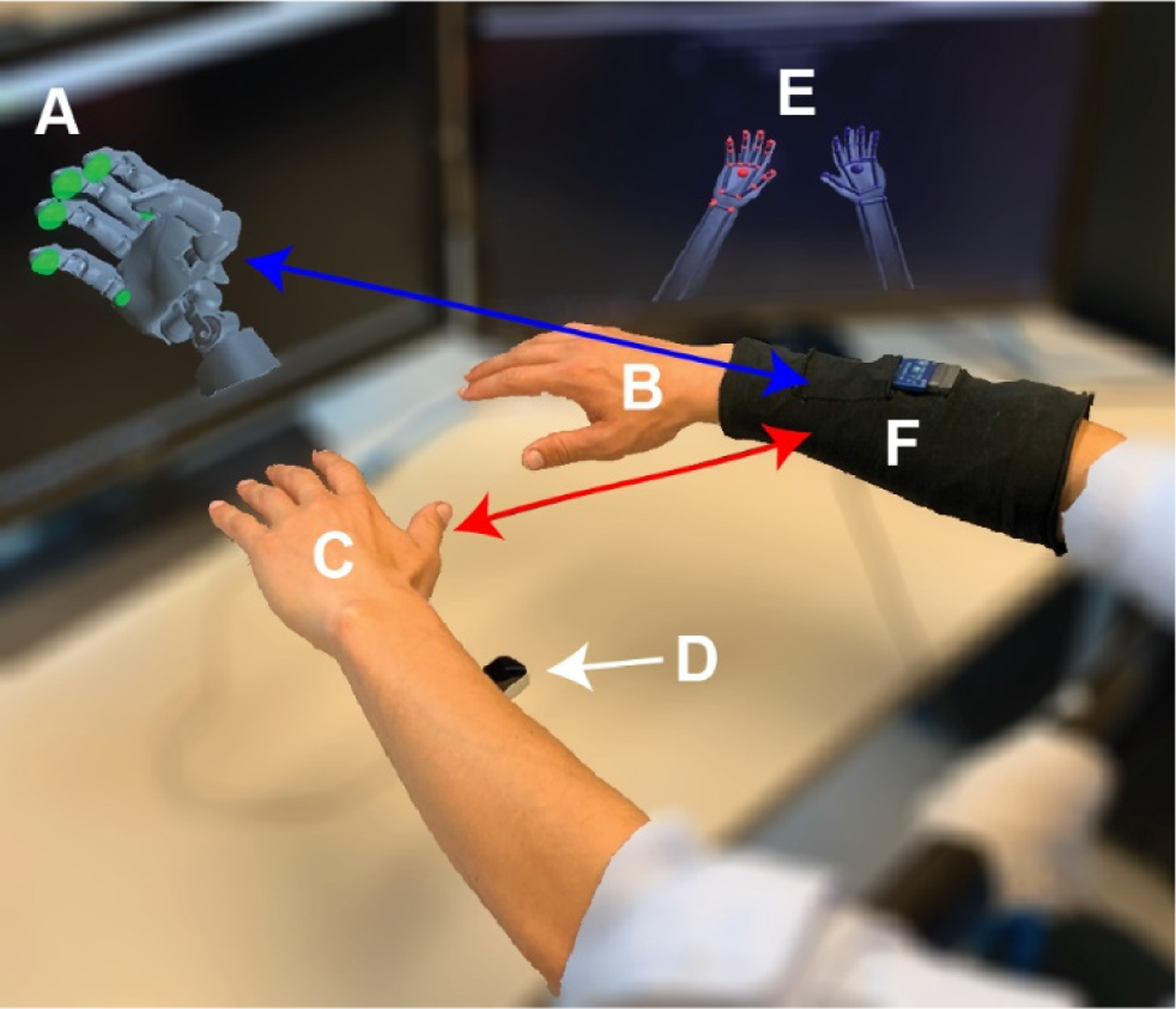
Data collection. Participants mimicked preprogrammed movements of a virtual hand (A) with their right hand (B) and left hand (C) simultaneously. A motion-capture camera (D) was used to measure the kinematics of both hands (E). The kinematics of the right hand serves as the ground-truth kinematics. The mirrored kinematics of the left hand serve as training data under mirror training (red arrow). The preprogrammed kinematics of the virtual hand serve as training data under mimic training (blue arrow). Differences between the kinematics of the right and left hands, and between the kinematics of the right and virtual hands, are used to assess the accuracy and precision of different training paradigms. EMG is recorded from the right forearm (F) and algorithms are trained under mimic training and mirror training. The predictions of those algorithms are then compared to the ground-truth kinematics from the right hand to assess the functional control afforded by mimic training and mirror training.

Seven healthy, non-amputee participants were instructed to mimic the movements of the virtual hand displayed on a computer monitor with their right hand and left hand simultaneously (Fig. 2). Each participant’s right hand served as the ground-truth kinematics, their left hand served as the mirror-training kinematics, and the preprogrammed kinematics of the virtual hand served as the mimic-training kinematics. The right hand served as the ground-truth kinematics, regardless of the participant’s hand dominance. The virtual hand was preprogrammed to make individuated flexion/extension of D1, D2, D3, D4, D5, as well as wrist flexion/extension, wrist pronation/supination, and D1 abduction/adduction. The virtual hand was also preprogrammed to make a power grasp (simultaneous flexion of D1–D5) and a full-hand opening (simultaneous extension of D1–D5). Altogether, this yields 18 unique movements. Each movement was performed 10 times before progressing onto the next movement, yielding a total of 180 trials. The total duration of each individual movement was 1.5 s (made up of a 0.7-s deviation away from the resting hand position, a 0.1-s hold-time at the position of maximum deviation, and then another 0.7-s deviation back to the original resting hand position). There was a 1-s intertrial interval to allow rest between successive trials. The preprogrammed movements were performed in synchrony with a metronome to ensure a consistent rhythm and to minimize participant reaction time. Participants were given an opportunity prior to data collection to familiarize themselves with the methodology and metronome.

#### 2) Offline Machine-Learning Analysis With Healthy Participants:

To ensure there was enough data for training, validation, and testing of the machine learning algorithms, six additional participants were recruited to complete a modified version of the protocol described above. Similarly, the healthy, non-amputee participants were instructed to mimic the movements of the virtual hand displayed on a computer monitor with their right hand and left hand simultaneously (Fig. 2). This time however, the virtual hand was preprogrammed to mimic the Degrees-Of-Freedom (DOFs) of a multi-articulate bionic arm (LUKE Arm, DEKA, Manchester, New Hampshire, USA). The virtual hand was preprogrammed to make individuated flexion/extension of D1, D2, D3, as well as wrist flexion/extension, wrist pronation/supination, and D1 abduction/adduction. No combination movements were performed. This reduced the total number of movements to 14, thereby allowing the participant’s time to be reallocated to collecting additional trials of each movement. Participants performed 20 trials of each movement, for a total of 280 trials. Participants then completed the procedure two additional times with a short break between each procedure to limit muscle fatigue. Altogether, a total of 840 trials were recorded from each participant.

#### 3) Online Prosthetic Control Analysis With Amputee Participants:

To compare the prosthetic control afforded by mimic training and mirror training, four participants with unilateral transradial amputations were recruited to complete the Clothespin Relocation Task (CRT) with a myoelectric prosthesis. Participants were age 56 ± 2.55 (mean ± standard deviation) and 75% male. The participants were instructed to perform the CRT with their intact hand to understand what movements would be necessary to complete the task. Participants then performed a brief session of mirror training and a brief session of mimic training to familiarize themselves with the training paradigms. Participants then donned the prosthesis (LUKE Arm, DEKA, Manchester, New Hampshire, USA) and performed a session of mirror training at their own pace using self-selected movement patterns they felt would be appropriate for the CRT. Participants were instructed that the prosthesis would only perform two movements: open/close of the hand (simultaneous flexion/extension of D1–D5) and pronation/supination of the wrist. Participants generally opted to perform simultaneous movements of both the hand and wrist during the self-directed mirror training. The training data from this first mirror-training session was then used to train an algorithm, and participants were allowed to temporarily control the prosthesis. The participants then performed a second self-directed mirror-training session to allow them to modify their self-directed motions and ensure viable control. The same two-stage training process is used for mimic training. To mitigate order effect, half the participants completed mirror training first and the other half completed mimic training first.

### D. Machine-Learning (Regression) Algorithms

Two different machine-learning algorithms were used in this study to see how different training paradigms impacted simple linear algorithms and more complex, non-linear algorithms. The first algorithm was a Kalman filter (KF), as described in [7]. The KF provides an efficient recursive algorithm to optimally estimate the position of the bionic hand when the likelihood model (i.e., the probability of EMG activity given the current kinematic position) and prior models (i.e., the state model of how kinematics change over time) are linear and Gaussian [27]. The inclusion of prior information about the system state enables an efficient recursive formulation of the machine-learning algorithm and effectively smooths noisy estimates in a mathematically principled way [27]. For online control, a Modified Kalman Filter (MKF) was used. The modification affects state behavior through gain and threshold values that edit the state between iterations during online use. No modifications to the gain were used in this study, but a 20% threshold was used, such that any kinematic predictions below 20% of the full kinematic range were set to zero, and anything above 20% was rescale between 0 and 100% of the full kinematic range.

The second algorithm used in this study was an eight-layer convolutional neural network (CNN). The CNN predicts kinematic position based on a spatiotemporal “image” of sEMG activity over the last 10 samples in time, described in more detail in [3]. The CNN utilizes convolution to learn complex spatiotemporal relations within EMG activity that correlate to kinematic position. For offline control, the CNN was trained with a learning rate of 0.0001 and 5 trials of the training dataset were used for validation. For online control, the CNN was trained with a learning rate of 0.001 and 10% of the training dataset was used for validation.

### E. Clothespin Relocation Task

The Clothespin Relocation Task (CRT) provides a simple way to assess the ability of individuals to simultaneously grasp and rotate their wrist. The CRT involves moving one clothespin per attempt from a horizontal bar to a vertical bar. Clothespins start 8 inches from the intersection of the vertical and horizontal bar along the horizontal bar and are placed by the participant 8 inches up the vertical bar. If the participant drops the clothespin or takes longer than one minute the attempt is considered a failure. To initiate and conclude each trial, participants were required to press a prominently sized button using the prosthesis. Successful execution of the task involved the prosthetic hand opening sufficiently to accommodate the clothespin between D2–4 and D1. This was followed by securely grasping the clothespin and transferring it from the horizontal bar to the vertical bar. The participant would then press the timer again to conclude the trial.

Participants were instructed to complete the CRT with the prosthesis under four different conditions: 1) using the CNN trained with data collected via mirror training, 2) using the CNN trained with data collected via mimic training, 3) using the MKF trained with data collected via mirror training, 4) using the MKF trained with data collected via mimic training. Participants performed the task six times for each of the four conditions. The four conditions were tested in pseudo-randomized counter-balanced blocks to minimize order effects. During each block, participants were given eight attempts to move the clothespins. A block was finished after three successful transfers or if all eight attempts were made. After the final block for a given condition, the participants completed the NASA Task Load Index (TLX) survey [28] of subjective workload as well as a survey of embodiment adapted from [29]. At the end of the experiment, participants were asked to rate the four control algorithms from best to worst. One participant had to end the experiment early and was unable to complete all TLX and embodiment surveys. As such, the TLX and embodiment data only captures three of the four participants.

### F. Data Analyses

#### 1) Biomechanical Analysis With Healthy Participants:

Mimic training requires the user to move a target DOF while keeping the other DOFs at rest. For most individuals, moving certain digits may cause movement in other digits either due to biomechanical coupling or user error. This collective phenomenon is referred to as *biomechanical coupling* in this study. To quantify biomechanical coupling during training movements, we measured the deviation from rest during each time point during training movements for non-target DOFs.

During mimic training, there are periods of rest in between movements in which the participant resets their hand back to a common resting position. It is unknown if participants can consistently recreate the same rest position in between trials as the mimic training paradigm assumes. We define the change in rest position throughout training as *drift*. To quantify drift, we measured deviation from rest for all DOFs during rest periods between movements and trials.

The mean for biomechanical coupling and drift were calculated across DOFs, and the median was taken from each participant. One sample t-tests were performed to determine if the data were significantly different from zero.

Additionally, we assessed spatial and temporal accuracy and precision as participants completed the training movements. We quantified spatial accuracy as the difference in peak magnitude of the attempted hand movements (e.g., reaching only 90% of the total range of motion instead of 100% of the total range of motion would yield a 10% magnitude error). We quantified spatial precision as the variance of peak magnitudes. We quantified temporal accuracy as the difference in time at which the peak magnitudes occurred. We quantified temporal prevision as the variance of the difference in time at which the peak magnitudes occurred. Importantly, the spatial and temporal accuracy and precision were calculated per participant. For example, for each participant we calculated the time delay associated with each of their individual movements. We then report the average time delay for this participant as one data point representing the accuracy of the method. We also report the variance of the time delays for this participant as one data point representing the precision of the method. This approach allows us to access precision at the individual level (i.e., how well the method captures differences in time delay among movements within a single individual) rather than at the population level (i.e., how well the method captures differences in time delay among individuals). Lastly, we also calculated the overall spatiotemporal accuracy as the root mean squared error (RMSE) of the two signals. Paired t-tests were used to compare the means of all of these metrics between mimicked training and mirrored training.

#### 2) Offline Machine-Learning Analysis With Healthy Participants:

For each training condition and algorithm, 10-fold validation was used for offline analysis of machine learning performance. To minimize any effects of order that may have appeared in the data, an equal number of trials were used from each of the three training sessions. The trials were “shuffled” so the testing data and training data varied from each training session. For the CNN, five trials were used for validation. RMSE was calculated for each time point of data and a median value was selected from each DOF. The median of the eight DOFs was selected to represent the performance for that analysis. The median number of the 10 analyses was used to represent each condition per participant. We performed this analysis from 10 to 55 out of the 60 total available training trials in increments of five for a total of 10 training dataset sizes. The remaining trials were used for testing (e.g., training with 10 trials resulted in 50 trials for testing). The resulting pooled data were screened for normality. A three-way analysis of variance (factors: training paradigm, algorithm, and dataset size) was performed, followed by subsequent post-hoc pairwise t-tests of the extreme conditions.

#### 3) Online Prosthetic Control Analysis With Amputee Participants:

Data were screened for normality. A two-way analysis of variance (factors: algorithm and training paradigm) was performed separately for each outcome metric. No significant interaction was observed, so the model was rerun for strictly main effects. In cases where the data were non-parametric, a Kruskal-Wallis was performed for the main effects. Because the number of completed clothespin transfers varied based on success rate and was nonparametric, a Wilcoxon rank-sum test was used to compare the time to completion between mimicked training and mirrored training.

## III. Results

### A. Preprogrammed Movements of a Virtual Hand Did Not Account for Biomechanical Coupling or Temporal Changes in Resting Hand Position

**Fig. 3.**
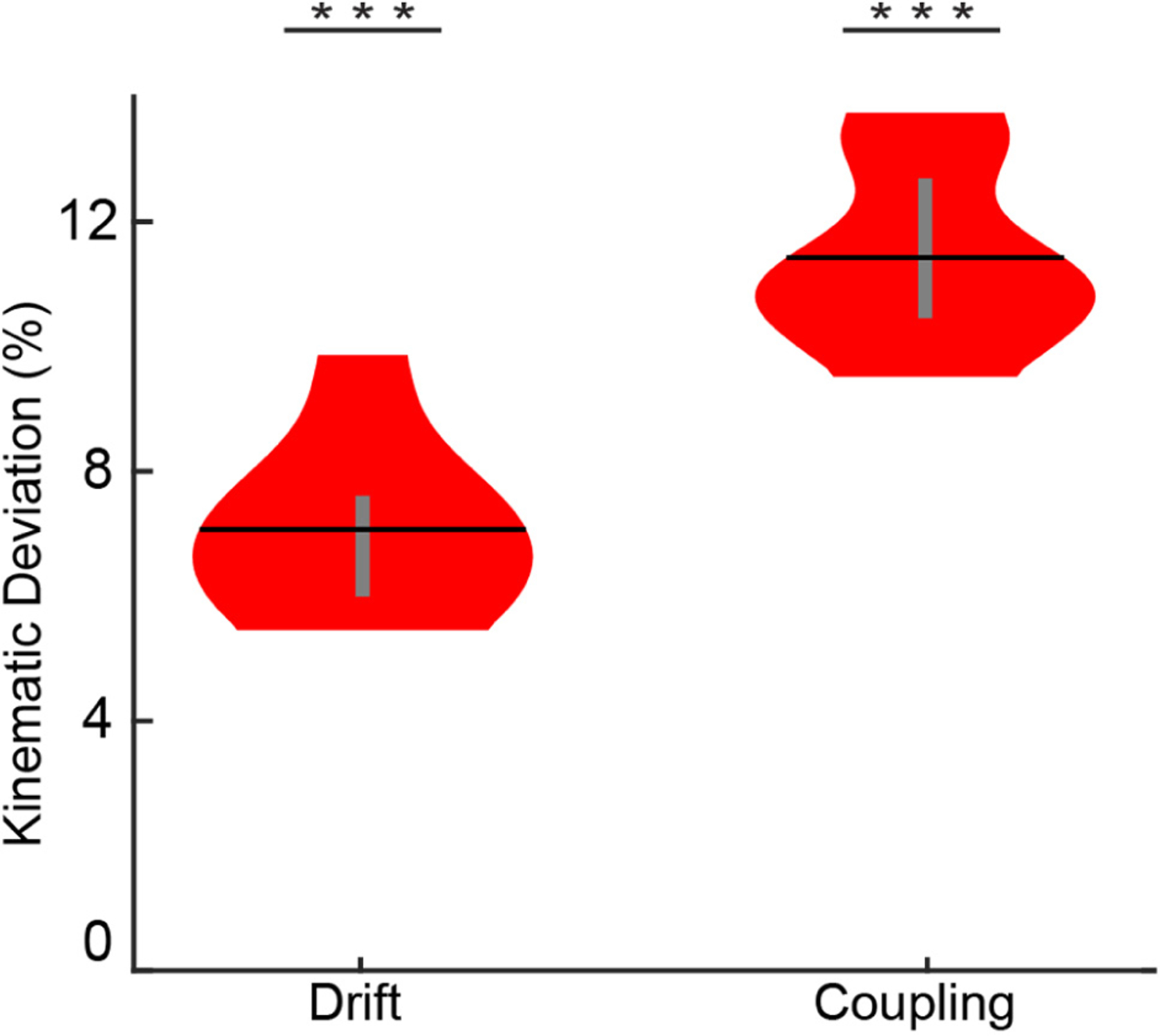
Deviation in natural hand kinematics when mimicking a virtual hand. Although a virtual hand makes perfectly consistent and perfectly isolated movements, the natural hand cannot. There are significant, non-zero kinematic deviations due to biomechanical coupling and temporal changes in resting hand position. Violin plots show the kernel density estimation. Black horizontal lines denote the mean and vertical gray lines denote the interquartile range. *** *p* < 0.001, one-sample t-test.

Mimic training assumes that the user performs perfectly identical and isolated kinematic motions while mimicking the prosthesis, which seems unlikely given the natural variance of hand motions. To explicitly test the validity of this assumption, we quantified the biomechanical coupling and temporal changes in resting hand position associated with an individual’s hand as they attempted to mimic a virtual hand. We found that the human hand had significant biomechanical coupling (*p* < 0.001, one-sample t-test) and significant temporal changes in resting hand position (*p* < 0.001, one-sample t-test). Biomechanical coupling resulted in an 11.43 ± 0.57% (mean ± S.E.M.) kinematic deviation and resting-hand-position drift resulted in a 7.07 ± 0.56% kinematic deviation (Fig. 3). Thus, the assumption that an individual can perform perfectly identical and isolated kinematic motions is invalid.

### B. Mirroring Contralateral Movements Reduced the Error and Variability of Movement Magnitude

**Fig. 4.**
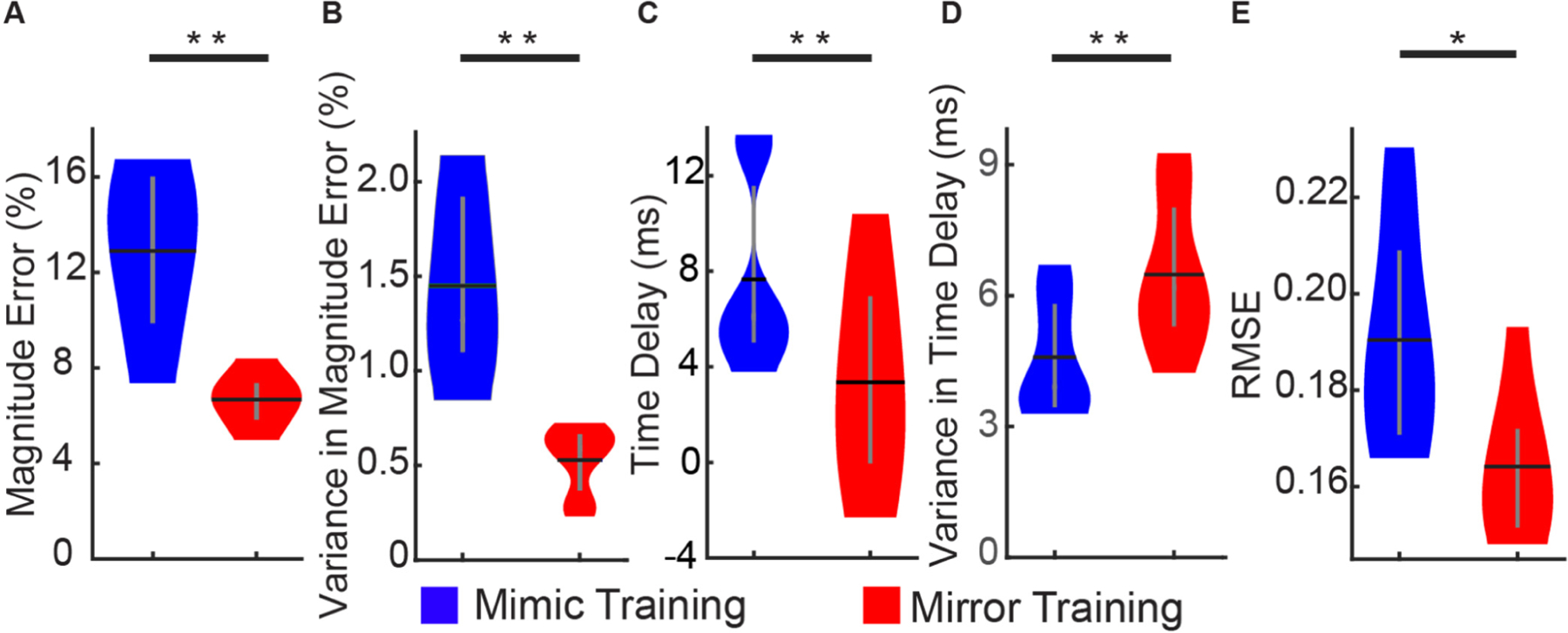
Accuracy and precision of mimic and mirror training. A) For the error associated with the magnitude of movements, mirror training was more accurate (less error). B) Mirror training was also more precise (less variation in errors within a participant). C) For the error associated with the timing of movements, mirror training was more accurate (less delay). D) However, mirror training was less precise (more variation in delays within a participant), likely due to the fact that the hand is consistently delayed when mimicking, whereas the hand can both precede and lag when mirroring. E) Overall, the RMSE associated with mirror training was significantly less than that associated with mimic training. Data from seven healthy, non-amputee participants. Violin plots show the kernel density estimation. Black horizontal lines denote the mean and vertical gray lines denote the interquartile range. * *p* < 0.05, ** *p* < 0.01, paired t-test.

Mirror training assumes that an individual can more accurately and precisely mirror their own contralateral hand than they can mirror the preprogrammed motion of a prosthesis. To explicitly test the validity of this assumption, we had healthy non-amputee participants perform mirror training and mimic training at the same time, such that we could see how closely the kinematics of their hand resembled the kinematics of their contralateral hand and the kinematics of the virtual hand they were mimicking. We found that under mirror training, training data more closely captured the magnitude of individual movements. That is, the error associated with the maximum joint angle achieved during a movement (i.e., magnitude error) was significantly less with mirror training than with mimic training (6.67 ± 0.42% vs 12.89 ± 1.35%; *p*< 0.005, paired t-test; Fig. 4A). Furthermore, the variance of the magnitude errors was also significantly less with mirror training than with mimic training (0.53 ± 0.07% vs 1.45 ± 0.18%; *p* < 0.005, paired t-test; Fig. 4B). Thus, when considering the range of motion associated with movements, an individual can more accurately and precisely mirror their own contralateral hand than mimic a virtual hand.

### C. Mirroring Contralateral Movements Reduced the Error in Movement Timing but Increased the Variability of Timing Errors

Under mimic training, there is a reaction-time delay between the preprogrammed kinematics of the virtual hand and the user’s kinematics and associated muscle activity. The assumption that an individual can more accurately and precisely mirror their own contralateral hand than a virtual hand is partly founded on the fact that there is no reaction-time delay during bilaterally mirrored movements. Using the experimental paradigm described above, we explicitly tested the validity of this assumption as well. We found that errors in the timing of movements were indeed significantly less for mirror training than for mimic training (3.35 ± 1.73 ms vs 7.66 ± 1.53 ms; *p* < 0.05, paired t-test; Fig. 4C). However, the variance of timing errors was significantly greater for mirror training than for mimic training (6.48 ± 0.68 ms vs 4.59 ± 0.51 ms; *p* < 0.005, paired t-test; Fig. 4D). Thus, although a user can more accurately time the motion of their hand with that of their contralateral hand, they cannot do so more precisely. In other words, the delay due to reaction time is larger, but more consistent than the delay between bilaterally mirrored movements.

### D. Overall, Mirror Training Yielded More Accurate Training Data Than Did Mimic Training

Mirror training had significantly less error associated with the magnitude and timing of movements. In addition, we found that the RMSE between the ground-truth kinematics and the contralateral hand (i.e., mirror training) was significantly lower than the RMSE between ground-truth kinematics and the preprogrammed movement of the virtual hand (i.e., mimic training). Mirror training had an RMSE of 0.16 ± 0.01% while mimic training had an RMSE of 0.19 ± 0.01% (*p* < 0.05, paired t-test; Fig. 4E). Thus, overall, the assumption that an individual can more accurately mirror their own contralateral hand than a virtual hand is true.

### E. Mirror Training Improved Offline Performance of Myoelectric-Control Algorithms When There Was a Sufficiently Large Amount of Data

**Fig. 5.**
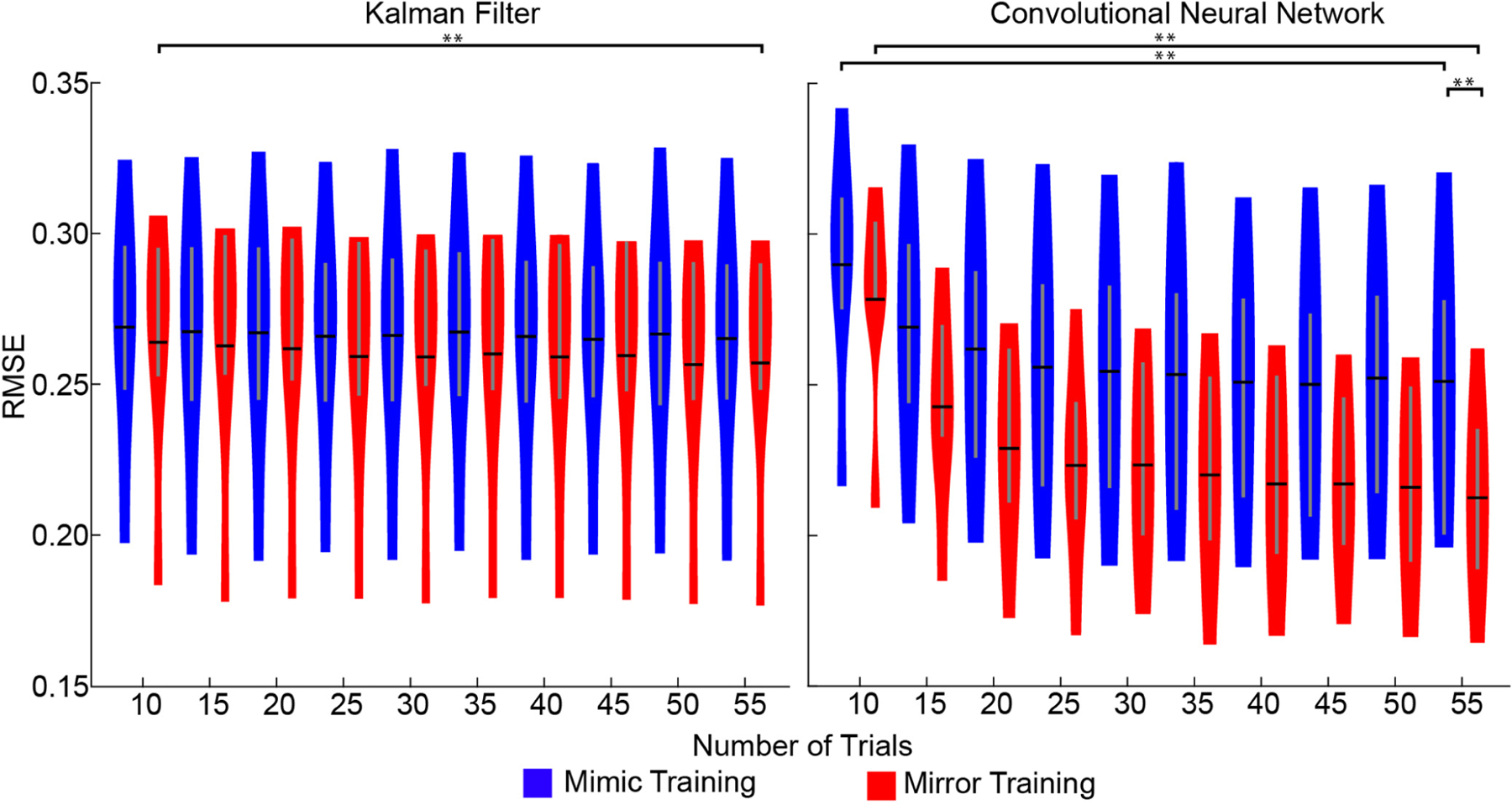
Offline performance of myoelectric control algorithms with increasing data and different training paradigms. When the training dataset is small (i.e., 10 trials), there is no significant difference between mimic or mirror training. This is true for both a linear Kalman filter (left) and a non-linear convolutional neural network (CNN; right). However, when the training dataset is large (i.e., 55 trials), mirror training yields significantly better performance for the CNN, but not for the Kalman filter. Increasing the size of the dataset (from 10 trials to 55 trials), significantly improved the performance of the CNN for both mimic and mirror training, but the improvement seen was greater under mirror training. In contrast, the performance of the Kalman filter improved with the larger dataset only when using mirror training, and that improvement was slight. Data from six healthy, non-amputee participants. Violin plots show the kernel density estimation. Black horizontal lines denote the mean and vertical gray lines denote the interquartile range. ** *p* < 0.005, paired t-test.

Having assessed the accuracy and precision of the underlying training data, we next sought to explore how the accuracy and precision of training data impact the performance of myoelectric control algorithms. To do this, we trained both a linear Kalman filter and a non-linear CNN under both mirror and mimic training. Initially, we trained the algorithms using a modest dataset consisting of 10 trials of each movement and found no significant difference between mirror or mimic training for either of the algorithms (*p* = 0.15 and *p* = 0.46, paired t-tests; Fig. 5). Knowing that mirror training generates a more faithful, but more complex training dataset, we hypothesized that a larger dataset would be needed to reveal the benefits of mirror training. To this end, we then trained the algorithms using increasingly larger datasets. A three-way analysis of variance (factors: training paradigm, algorithm, dataset size) revealed a significant effect for dataset size (*p* < 0.01), a significant interaction between training paradigm and algorithm (*p* < 0.05), and a significant interaction between dataset size and algorithm (*p* < 0.05). Post-hoc pairwise comparisons between the smallest dataset size (10 training trials) and the largest dataset size (55 training trials) revealed that increasing dataset size significantly improved the performance of the CNN, and this was true for both mirror and mimic training (*p*’s < 0.01, paired t-tests; Fig. 5). For the Kalman filter, we found that increasing the amount of data significantly improved performance with mirror training (*p*< 0.01, paired t-test). A similar but non-significant effect was seen with mimic training (*p* = 0.0618, paired t-test; Fig. 5). Most importantly, with this large dataset, we found that mirror training significantly outperformed mimic training when using the CNN (*p* < 0.01, paired t-test) but not when using the Kalman filter (*p* = 0.25, paired t-test; Fig. 5). Thus, more accurate and more precise training data can improve the performance of myoelectric control algorithms, but only when there is sufficient data and when using a more complex, non-linear algorithm.

### F. Mirror Training Improved Task Completion Speed for Transradial Amputees Performing the Clothespin Relocation Test of Upper-Limb Dexterity

One limitation of the above offline analyses is that both mimic and mirror training were performed simultaneously, making it difficult to disentangle the user’s emphasis on one approach over the other. To this end, we next explored whether the offline improvements in algorithm RMSE would translate to meaningful improvements on functional tasks when the user is actively in the loop and completes the trainings independently as they would in a real-world scenario.

**Fig. 6.**
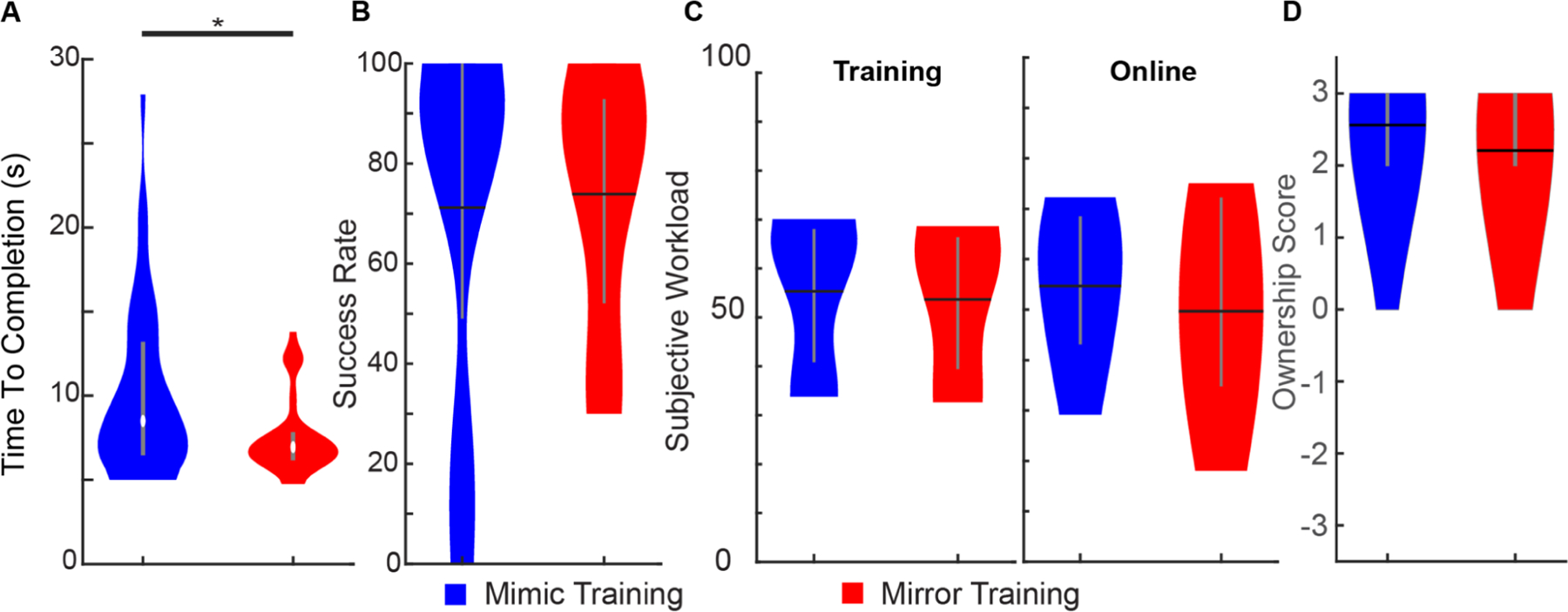
Performance of amputee participants completing a clothespin relocation task (CRT) with a myoelectric prosthesis trained with mirror or mimic training. A) Participants complete the CRT faster with mirror training. B) Success rate was similar for mirror and mimic training (out of 39 attempts for mimic training and out of 42 attempts for mirror training). C) The cognitive load associated with mirror and mimic training is also similar both during training and online use. D) There were also no significant differences between embodiment scores associated with mirror and mimic training. Data from four unilateral transradial amputees for A and B. Data from three unilateral transradial amputees for C & D. Violin plots show the kernel density estimation. Black horizontal lines denote the mean, white circles denote the median, and vertical gray lines denote the interquartile range. * *p* < 0.05, Wilcoxon rank-sum test.

To this end, we recruited four unilateral transradial amputees to complete the clothespin relocation test using mirror or mimic training in a cross-over study. We found that mirror training significantly improved task competition speed (6.95 ± 1.60 s for mirror training vs 8.50 ± 6.67 s for mimic training, median ± IQR; *p* < 0.05, Wilcoxon rank-sum; Fig. 6A). No significant effects were observed in terms of the success rate or the control algorithm (Fig. 6B). Additionally, there were no significant differences regarding the subjective workload or embodiment scores between the two training paradigms (Fig. 6C and 6D). User preference among the training paradigms varied; participants two and four favored mirror training, participant one favored mimic training, participant three had no preference.

## IV. Discussion

In this study, we explored the validity and impact of two common training paradigms for myoelectric prosthesis: mimic training and mirror training. We found that mirror training produces a training dataset that more accurately represents the complex kinematics of natural hand movements. However, we also found that the benefits of this more accurate dataset were realized only with large datasets and more complex algorithms. Lastly, we showed that holistically mirror training can also improve the function of transradial prostheses for physical tasks. Together, these findings demonstrate that the myoelectric training paradigm can directly influence the algorithm and user performance. These findings also support the use of mirror training over mimic training where possible.

Historically, mimic training has been the most used myoelectric training paradigm. One contributing factor is that some amputees suffer from bilateral amputations and would not be able to perform mirror training. Another contributing factor is likely the cost and complicated setup associated with motion capture systems. Indeed, optical, marker-based motion capture can cost tens of thousands of dollars and require multiple hours of setup. The advent of markerless motion capture with low-cost cameras [30], [31], [32] has only recently made mirror training possible in unsupervised home environments. Although the accuracy of these low-cost markerless systems is not perfect [33], [34], they can provide real-world functional benefits to patients using myoelectric prostheses, as demonstrated herein.

Neurophysiological studies on the neural basis of movement support the observed benefits of mirror training. When mirroring movements, there’s additional neural activation [35], [36], [37], that may enhance cortico-muscular responsiveness [38], [39], [40]. Indeed, it has been suggested that a common neural drive exists for bilaterally mirrored movements [41]. Although distinct, there is substantial overlap among the neural pathways associated with unilateral and bilateral movements, which has been shown to facilitate motor learning [42]. Illusory movement through mirror therapy has also been shown to counteract learned non-use of the limb [43]. Together, these factors may enhance an individual’s motor learning, accuracy, and precision, which in turn yields improved prosthesis control.

These insights support the idea that mirror training enhances both temporal and spatial coordination. However, we note that the advantages of mirror training appear to primarily originate from its ability to improve spatial alignment rather than temporal alignment. In other words, the benefits of mirror training are not simply due to eliminating the reaction time delay associated with mimic training. When training algorithms under mimic training, reaction time delay is accounted for with a metronome/prompting and by temporally aligning the kinematic and EMG data. Consistent with prior work [3], [7], in this study we used a cross-correlation algorithm to eliminate reaction time delay. Furthermore, we report here that mirror training has greater variability in timing errors relative to mimic training. That is, the delay due to reaction time is more consistent than the delay between bilaterally mirrored movements. Further analysis of the temporal delays highlighted that with mimic training the user always lagged the preprogrammed kinematics. In contrast, with mirror training, the user both preceded and lagged their contralateral hand. The increased temporal variability suggests that simple cross-correlation approaches may not be appropriate for aligning training data collected under mirror training, and that new trial-by-trial realignment methods [23] are likely required for mirror training. Thus, after correcting for reaction time, mimic training tends to provide greater temporal alignment than mirror training, suggesting that the observed benefits more likely stem from improved spatial alignment.

Mirror training also stands out for its capacity to allow patients to perform self-selected motions more readily. As an example, during online use of the prosthesis, the amputee participants self-selected to perform simultaneous movements of both DOFs. In contrast, the preprogrammed movements during mimic training were performed for each DOF in isolation. Preprogramming natural coordinated motions across multiple DOFs for use in mimic training would be complex and prohibitive.

Mirror training, therefore, offers a more efficient approach, allowing participants to focus selectively on the most relevant combinations and sequences tailored to the specific task at hand. This circumvents the need for extensive programming or selection of movements. Although it’s possible to preprogram any arbitrary motion for mimic training, mirror training allows users to quickly generate custom training datasets that more accurately represent natural hand kinematics and that fit user preferences. To this end, we also observed that participants changed their self-selected movements between the first and second mirror training sessions to improve their control.

As noted above, we also observed that participants self-selected to perform combination movements (e.g., grasping while rotating) rather than individuated movements. On this point, our work is consistent with that of [4], which showed greater performance on the clothespin relocation task when using training data consisting of combination movements instead of individuated movements. Future work should explore the differences between mimic and mirror training when combination movements are performed for both training paradigms.

This study focused strictly on regression algorithms; future work should explore if the benefits of mirror training translate to classification algorithms. We hypothesize that the benefits of mirror training may be less profound for classification algorithms, as classification approaches simply differentiate among discrete classes and make predictions across a longer timeframe (i.e., after completion of a gesture) [44], [45]. As such, intraclass variability is less of a concern when there is large separation among classes. Nevertheless, as more gestures are added, and the interclass separation lessens, mirror training may offer an opportunity to minimize intraclass variability. More broadly, regression algorithms have been shown to outperform classification approaches in functional tasks [46]. As regression control becomes more widespread, this study emphasizes the importance of training data, as subtle changes in the training data can lead to significant differences in runtime performance.

In the present study, the prosthesis control algorithms were trained in a biomimetic way, such that the users intended kinematics were consistent with the prosthesis action. By example, when the user attempted a grasp, the algorithm was trained such that the prosthesis would perform a grasp. An alternative approach is non-biomimetic control, in which algorithms are trained to map arbitrary muscle patterns to arbitrary prosthesis actions. Non-biomimetic control offers the ability to have the user perform specific muscle patterns that minimize intraclass variability and maximize interclass separability, and has been shown to improve functional outcomes [47]. Future work should investigate how mimic and mirror training could be best utilized within the framework of non-biomimetic control strategies. Future work should also explore the impact of mirror and mimic training for various patient characteristics, including, for example, hand dominance, sex, time since amputation, and level of amputation.

## V. Conclusion

Supervised machine learning approaches have emerged as the most popular algorithms for more dexterous myoelectric and neural prostheses. Although the field has spent considerable time exploring more advanced machine-learning algorithms, little attention has been paid to ensure the highest quality training data. In this study, we assessed the validity of the assumptions underlying common training paradigms and quantified the accuracy and precision of the training data. We also demonstrated that the selection of a particular training paradigm can significantly impact the performance of the myoelectric control algorithm and the amputee end-user. These findings support the use of mirror training for myoelectric control and reemphasize the importance of the training data in applications of machine learning.
